# Validation of a simple binary scoring system for assessment of welfare measures of 10-day-old commercial broilers and their correlation with environmental parameters

**DOI:** 10.1186/s40781-015-0039-3

**Published:** 2015-03-07

**Authors:** Priyanka Kumari, Hong-Lim Choi, Shamira Hazi Metali, Siti Anisah Hazi Yussof, Jiwoon Han

**Affiliations:** Department of Agricultural Biotechnology, Research Institute for Agricultural and Life Science, Seoul National University, Seoul, 151-742 Republic of Korea; Department of Biology, Faculty of Science, University of Brunei, Darussalam, Brunei

**Keywords:** Airborne microbes, Air temperature, Environmental parameters, Welfare measures

## Abstract

**Background:**

A simple binary scoring system (SBSS) was developed and used to assess the welfare measures of commercial broiler chickens in South Korea. We also correlated welfare measures with environmental parameters of broiler house. Our measures of welfare included lameness, hock burn (HB) and foot pad dermatitis (FPD), whilst environmental parameters included air temperature, relative humidity, air speed, light intensity, air quality (in particular carbon dioxide (CO_2_) and ammonia (NH_3_) concentrations) and airborne microbes.

**Results:**

The effect of environmental parameters on welfare measures was apparent even on 10-day-old broilers. A non-parametric correlation analysis revealed significant correlations between environmental parameters and welfare measures. The key environmental parameters were relative humidity and light intensity. The results indicate that there is a need for proper control of environmental conditions on poultry farms, which could reduce health problems and subsequently reduce disease and mortality.

**Conclusions:**

In conclusion, the simplicity of SBSS makes it preferable over more complex scoring systems and allows a farmer to more easily assess the welfare measures on their own farm.

## Background

Due to their short reproductive cycle and worldwide popularity as food animal production, poultry generally represent the most-highly selected type of livestock. The selection of broiler chickens has been primarily directed at economic specialty, which has reduced the costs of production [1]. Throughout the world, the majority of broilers are reared in intensive production systems, where birds are confined for their lifetime under a high density stocking environment [2]. They are reared from hatch to slaughter and weighed monthly. It may take less than 40 days to go from hatch to slaughter.

Because animal welfare assurance has become an important aspect of the marketing of poultry products to food retailers, the need has arisen for an appropriate method to measure welfare in commercial broiler flocks. Visual inspection of welfare measures offers the advantage of allowing a noninvasive evaluation of a large number of birds in a short period of time. A valid and feasible method for scoring broiler welfare on commercial farms is needed so that farmers can easily apply a consistent scoring method in a reasonable length of time. Presently, Welfare Quality assessment protocol of European Union has been used to assess welfare measures in breeder stocks and has been adapted for the evaluation of commercial broiler production [3]. Welfare Quality assessment protocol defines 3 to 6 scoring categories on an ordinal scale of severity. The differences between categories are subtle enough to make scoring perhaps more difficult and slower than necessary in a commercial production environment. Therefore there is a need to develop a simple welfare assessment protocol, which will be simple enough for routine use under commercial conditions.

The internal environment of poultry buildings is a complex dynamic system influenced by many contributory factors. A number of these factors impact bird health, behavior and productivity. Environmental conditions are generally considered as the main factors affecting the welfare of broilers in farms. Environmental conditions that are thought crucial to broiler welfare include: temperature, humidity, light intensity, air speed and air quality [4-8]. Broilers release many harmful substances from their metabolism and various activities, such as carbon dioxide (CO_2_), ammonia (NH_3_) and airborne microbes [9-11]. To keep the broilers and workers healthy, the concentration of these pollutants in air should be monitored and reduced to safe limits.

A real-time simple binary scoring system (SBSS), which seeks to identify broilers as having no impairment (score 0) and having severe impairment (score 1). As discussed above, the present study aimed to test the effectiveness of SBSS by evaluating the welfare measures (lameness, hock burn (HB) and foot pad dermatitis (FPD) of commercial broilers in South Korea. We also assessed the impact of environmental parameters (air temperature, relative humidity, air speed, light intensity, air quality and airborne microbes) on these welfare measures of commercial broilers.

## Methods

### Site selection and experimental design

The procedures in this study were carried out in accordance with the accepted ethical standards and all the welfare parameters were measured carefully with proper hygienic maintenance. The study was conducted over a spring period (March to June, 2013) in South Korea. The broiler flocks studied were on a farm that grew chicken in a modern production system. Management and husbandry data were recorded for the house. This information included house and wall type, floor type, date of floor cleaning, temperature (air and body), total number of birds on site (at the time of visit), total number of birds originally placed in house, total number of birds in house (at the time of the visit), age at day of inspection, parent flock age(s), genotype, average bird weight at time of visit (taken from records of weights taken by the producer), feed quality and time interval, feed withdrawal rate, number of stock workers and number of visit per hours, vaccination for disease and time of vaccination, mortality/month, culls/month, when a month cull rate was high and why (throughout the year) and when a month mortality rate was high (Table 1).

**Table 1:** **Details of management and husbandry data from broiler house**

**General information**	**Results**
House type and wall type	H-Beam and sandwich panel
Floor type	Solid with bedding material was rice husks
Date of cleaning of floor	5/ 15/ 2013 (cleaning per month after sell)
Temperature (air and body)	33°C (air) and 39°C (body)
Total number of birds on site (at the time of visit)	22850
Total number of birds originally placed in house	23000 (total 90000 = 23000 + 17000 + 17000 + 17000 + 16000)
Total number of birds in house (at the time of the visit)	22850
Age at day of inspection	MD: 1 day, IB: 7 day, IBD: 14 day
Parent flock age(s)	10 day-old-bird
Genotype	Cobb broiler
Average bird weight at time of visit (taken from records of weights taken by the producer)	344 g (10 day after birth)
Feed quality and time interval	Feed bought from Jeil Feed company and feeding was operated automatically
Feed withdrawal rate	0
Number of stock workers	1
Vaccination for disease, Time of vaccination.	MD: 1 day, IB: 7 day, IBD: 14 day
Mortality/month	5%
Culls/month	2.5%
In which month calls rate is high and why (throughout the year)	November to February because of cold
In which month mortality rate is high and why (throughout the year)	November to February because of cold

### Sampling points and measurement of environmental parameters

All the welfare measures and environmental parameters were measured at nine points inside a chicken house at 15 cm above the floor, which corresponded to the nose height of the chickens and data was collected in triplicate from each point. The arithmetic means and SD of the each variable were calculated before further statistical analysis. Air temperature and relative humidity were measured with a hygrothermograph (SK-110TRH, SATO, Tokyo, Japan). Air speed was measured with an anemometer (model 6112, KANOMAX, Osaka, Japan). Light intensity (lux) values were recorded using a light meter (ANR-F9 LUX METER, Tokyo Photo-electric Co. Ltd, Japan) held at arm’s length and at bird height. The greenhouse gases concentrations in particular CO_2_ and NH_3_ were measured using a GASTECH device (Pump kit No. 101). A GASTECH device was used despite having several limitations because it is simple to handle and a typical farmer can easily identify the various compounds with the help of this instrument.

### Airborne bacterial analysis

Airborne bacterial counts were measured using a settle-plate method. This is a direct method for assessing the likely number of microorganisms depositing onto the product or surface in a given time. It is based on the fact that in the absence of any kind of influence, airborne microorganisms, typically attached to larger particles, will deposit onto open culture plates. Tryptic soy agar (Merck, Darmstadt, Germany) was used for enumeration of total airborne bacteria, and Chromocult Coliformen agar (Merck, Darmstadt, Germany) was used for Total Coliforms and *Escherichia coli*. After sampling, the plates were incubated at 37°C for 48 h, and the colonies were counted and calculated as colony-forming units. Samples were collected from nine points in triplicate.

### Assessment of welfare measures

In the field observations, two observers scored birds using the binary system in the commercial flock. Walking ability (lameness) and leg health was inspected and scored according to the definitions given in Table 2. Walking ability was recorded for 15 chickens at nine designated points enclosed in a small frame and when allowed to walk out one-by-one. The ease with which the birds walked was scored after observing nine walking areas. A further 15 chickens were examined for HB and FPD.

**Table 2:** **Scoring of lameness, foot pad dermatitis and hock burn**

**Welfare measures**	**Score**	
	**0**	**1**
Lameness	Absence of lameness	Presence of lameness
Foot pad dermatitis	No lesion	lesions
Hock burn	No discoloration or lesions	Hock with lesion

### Statistical analysis

The details of management, husbandry and environmental parameters data were evaluated under the guidelines of Ross Broiler Management Manual [12]. The statistical evaluation was carried out with an SPSS software package (SPSS Inc., Chicago, IL). Pairwise correlations (Kendall’s τ-b correlation coefficients) were calculated among environmental parameters and welfare measures.

## Results and discussion

### Environmental parameters in broiler house

The means of the environmental parameters in the broiler house are presented in Table 3. The mean values for air temperature, relative humidity, airspeed and light intensity were 32.6° C, 0.12 m/s, 43.5% and 7 lux, respectively (Table 3). The broiler farm was equipped with a mechanical ventilation system provided with fans (with slot inlet at a sidewall), which controls air temperature and relative humidity better than naturally ventilated systems or systems with fan-assisted drop-down ventilation, indicating a more effective mixing of air and flow over the birds. The mean air temperature and relative humidity in the house were within the recommended range [12].

**Table 3:** **Environmental parameters measured inside broiler house**

**Parameters**	**Mean**	**s.e.m.**	**Min.**	**Max.**
Air temperature (°C)	32.69	0.149	32.00	34.00
Air speed (m/s)	0.12	0.022	0.05	0.27
Relative humidity (%)	43.54	0.358	42.00	45.00
Light intensity (lux)	7.00	0.500	5.00	10.00
CO_2_ (ppm)	1755.56	72.860	1200.00	2000.00
NH_3_ (ppm)	4.00	0.289	3.00	5.00
TAB^1^ (CFU/m^3^)	277.11	4.733	260.00	296.00
TC^2^ (CFU/m^3^)	53.67	5.747	33.00	83.00
TE^3^ (CFU/m^3^)	7.22	4.784	0.00	34.00

^1^Total airborne bacteria; ^2^Total coliform; ^3^Total *E. coli*.

Jones et al. [4] emphasized the importance of temperature and relative humidity to the health and mortality of broiler chickens produced in a northern European climate by analyzing the temperature and humidity profiles throughout the growth cycle. Jones et al. [4] found that high temperature and relative humidity adversely affect the lameness, FPD and mortality. Air speed has also been shown to significantly affect broiler performance [8]. Deep et al. [13] showed that light intensity did not affect broiler production and mortality, but did affect carcass characteristics.

The mean concentrations of CO_2_, and NH_3_ were 1755.5 and 4.0 ppm, respectively, in the house within the farm across nine different points (Table 3). The concentrations of these gases were within the recommended range suggested for broiler houses [12]. CO_2_, originating from animal respiration as well as from manure breakdown, is an important gas in confined animal buildings. Concentrations and emissions of CO_2_ have sometimes been used to estimate poultry house ventilation rates [14,15]. High concentrations of NH_3_ inside animal houses represent potential health hazards to humans and animals [16]. The mean concentration of TAB, TC and TE in the broiler houses were 277.1, 53.6 and 7.2 (cfu/m^3^), respectively (Table 3). It has been shown that dead and partially decomposed air microbes may cause inflammation in the respiratory organs, and antigens and allergens may activate the immune system, leading to allergic reactions [17,18].

### Welfare measures of broiler house

The results of the welfare parameters evaluated from a broiler house at nine different points are presented in Table 3. Based on the mean score of the evaluation of the incidence and severity (degree) of welfare parameters (lameness, FPD and HB), it was found that most of the birds walking ability (lameness 0) was very good with mean score of 97% (Table 4). However, hock burn (hock 0) and footpad (footpad 0) conditions were very poor with mean score of 58.5% and 20.7%, respectively (Table 4).

**Table 4:** **Welfare measures evaluated from broiler house**

**Welfare measures**	**Mean**	**s.e.m.**	**Min.**	**Max.**
Lameness score 0 (%)	97.03	1.17	93.33	100.00
Lameness score 1 (%)	2.96	1.17	0.00	6.66
Hock score 0 (%)	58.51	6.06	33.33	86.66
Hock score 1 (%)	41.48	6.06	13.33	66.66
Footpad score 0 (%)	20.74	10.39	0.00	100.00
Footpad score 1 (%)	79.25	10.39	0.00	100.00

### Correlation between environmental parameters and welfare measures

The relationships between environmental parameters and welfare measures are presented in Table 5. The number of birds walking well (lameness 0) was negatively correlated with relative humidity (*P* < 0.05). Dawkins et al. [5] also found that the percentage of broilers with good walking ability (lameness 0) was negatively correlated with relative humidity. Furthermore, light intensity was found negatively correlation with foot pad dermatitis (FPD 0) (*P* < 0.01). Footpad dermatitis is caused by a multifactorial problem including various endogenous and exogenous factors [19]. Some of previous studies also showed that light intensity levels affect the footpad health of broilers [13,20]. Our results are in agreement with several other studies that have reported that environmental condition of the house is very crucial to bird welfare [4,5,8].

**Table 5:** **Kendall’s τ-b correlation coefficients for environmental parameters with welfare measures**

**Kendall’s τ-b**	**Lameness 0**	**Lameness score 1**	**Hock score 0**	**Hock score 1**	**Footpad score 0**	**Footpad score 1**
Air temperature	−0.572	0.572	0.216	−0.216	0.197	−0.197
Air speed	0.234	−0.234	−0.118	0.118	−0.500	0.500
Relative humidity	−0.756^*^	0.756^*^	0.457	−0.457	−0.121	0.121
Light intensity	−0.040	0.040	0.000	0.000	−0.839^**^	0.839^**^
CO_2_	0.000	0.000	−0.044	0.044	−0.093	0.093
NH_3_	−0.258	0.258	0.098	−0.098	−0.173	0.173
TAB	0.447	−0.447	0.028	−0.028	−0.030	0.030
TC	0.522	−0.522	−0.085	0.085	−0.030	0.030
TE	0.462	−0.462	−0.349	0.349	0.325	−0.325

*P < 0.5 (2-tailed); **P < 0.01 (2-tailed).

## Conclusions

The aim of our study was to apply the SBSS to assess the welfare measures of broilers and to evaluate the effect of environmental parameters on these welfare measures. Our results indicate that SBSS is a quick and simple scoring method for evaluating broiler welfare measures. This method could help in taking quick decisions about farm management maintenance issues, which could subsequently reduce disease and mortality and enhance broiler production. We also concluded that the control of the environment for broiler chickens is a key factor in improving their welfare, particularly through the control of relative humidity and light intensity.
